# A place for neutrophils in the beneficial pathogen-agnostic effects of the BCG vaccine

**DOI:** 10.1016/j.vaccine.2021.03.092

**Published:** 2021-04-15

**Authors:** Byron Brook, Frederick Schaltz-Buchholzer, Rym Ben-Othman, Tobias Kollmann, Nelly Amenyogbe

**Affiliations:** a Precision Vaccines Program, Boston Children’s Hospital, Boston, MA, USA; b Harvard Medical School, Boston, MA, USA; c Institute of Clinical Research, University of Southern Denmark and Odense University Hospital, Odense, Denmark; d Bandim Health Project, INDEPTH Network, Bissau, Guinea-Bissau; e Telethon Kids Institute, Perth, Western Australia, Australia

**Keywords:** BCG, Non-specific effects, Neutrophil

## Abstract

The BCG vaccine has long been recognized for reducing the risk to suffer from infectious diseases unrelated to its target disease, tuberculosis. Evidence from human trials demonstrate substantial reductions in all-cause mortality, especially in the first week of life. Observational studies have identified an association between BCG vaccination and reduced risk of respiratory infectious disease and clinical malaria later in childhood. The mechanistic basis for these pathogen-agnostic benefits, also known as beneficial non-specific effects (NSE) of BCG have been attributed to trained immunity, or epigenetic reprogramming of hematopoietic cells that give rise to innate immune cells responding more efficiently to a broad range of pathogens. Furthermore, within trained immunity, the focus so far has been on enhanced monocyte function. However, polymorphonuclear cells, namely neutrophils, are not only major constituents of the hematopoietic compartment but functionally as well as numerically represent a prominent component of the immune system. The beneficial NSEs of the BCG vaccine on newborn sepsis was recently demonstrated to be driven by a BCG-mediated numeric increase of neutrophils (emergency granulopoiesis (EG)). And experimental evidence in animal models suggest that BCG can modulate neutrophil function as well. Together, these findings suggest that neutrophils are crucial to at least the immediate beneficial NSE of the BCG vaccine. Efforts to uncover the full gamut of mechanisms underpinning the broad beneficial effects of BCG should therefore include neutrophils at the forefront.

## 1. Beneficial non-specific effects of neonatal BCG vaccination throughout childhood

The Bacille-Calmette-Guérin (BCG) vaccine, which is normally administered to prevent tuberculosis, has been demonstrated to reduce all-cause mortality and infectious-disease related morbidity far beyond tuberculosis [1]. This protective ability has been labeled off-target, heterologous or non-specific effects (NSE) [2,3]. Given that the host recognizes *Mycobacteria* via at least 18 innate pattern recognition receptors [4], and that innate immunity characteristically impacts a wide range of pathogens, this interaction might be better captured by the term ‘pathogen-agnostic’ effects [5]. For brevity we here will use the most widely used term, NSE.

Beneficial NSE of the BCG vaccine have most consistently been observed in the newborn period, where the mortality risk was reduced by 45% within three days of vaccination for low-birth-weight newborns who were randomized to receive BCG at birth [6,7]. This translates to a massive clinical impact, as nearly half of all <5 year deaths occur in the first week of life, with sepsis as the most common infection-related cause[8,9]. However, there is also strong evidence from both randomized clinical trials and observational studies that the BCG vaccine provides beneficial NSE beyond the immediate newborn period[10–15]

Non-specific effects include trained immunity, or durable long-lasting changes to innate immune cell function driven by epigenetic modification of progenitor cells [16]. However, NSE can also be achieved via transient, rapid immune responses to the live vaccine resulting in cellular proliferation in response to *Mycobacterim bovis* contained in the vaccine [17]. The search for mechanisms of NSE of vaccines has thus far focussed on altered monocyte function, a component of the longer-lasting trained immunity phenotype [2,16]. While evidence that BCG induces similar long-lived changes in neutrophil function are now starting to emerge [18], the neutrophil likely also plays a key role in the NSE immediately following administration of BCG. Due to their rapid response to environmental stimuli, short half-life [19], downregulation of genes from bone marrow-resident to circulating cells [20], and temperature- and physically-sensitive nature [21–23], neutrophils are inherently difficult to study. Yet, neutrophils are central to host immunity, evidenced by > 60% of human bone-marrow hematopoiesis dedicated to production of granulocytes [24]. Neutrophils furthermore exert vital roles in the immune responses to bacterial, fungal, and viral pathogens [25], as well as immunomodulatory functions that affect the behaviour of nearly all other host immune components [26].

## 2. Neutrophils and BCG-mediated NSEs against infectious disease

### 2.1. Sepsis in the newborn period

**Fig. 1.**
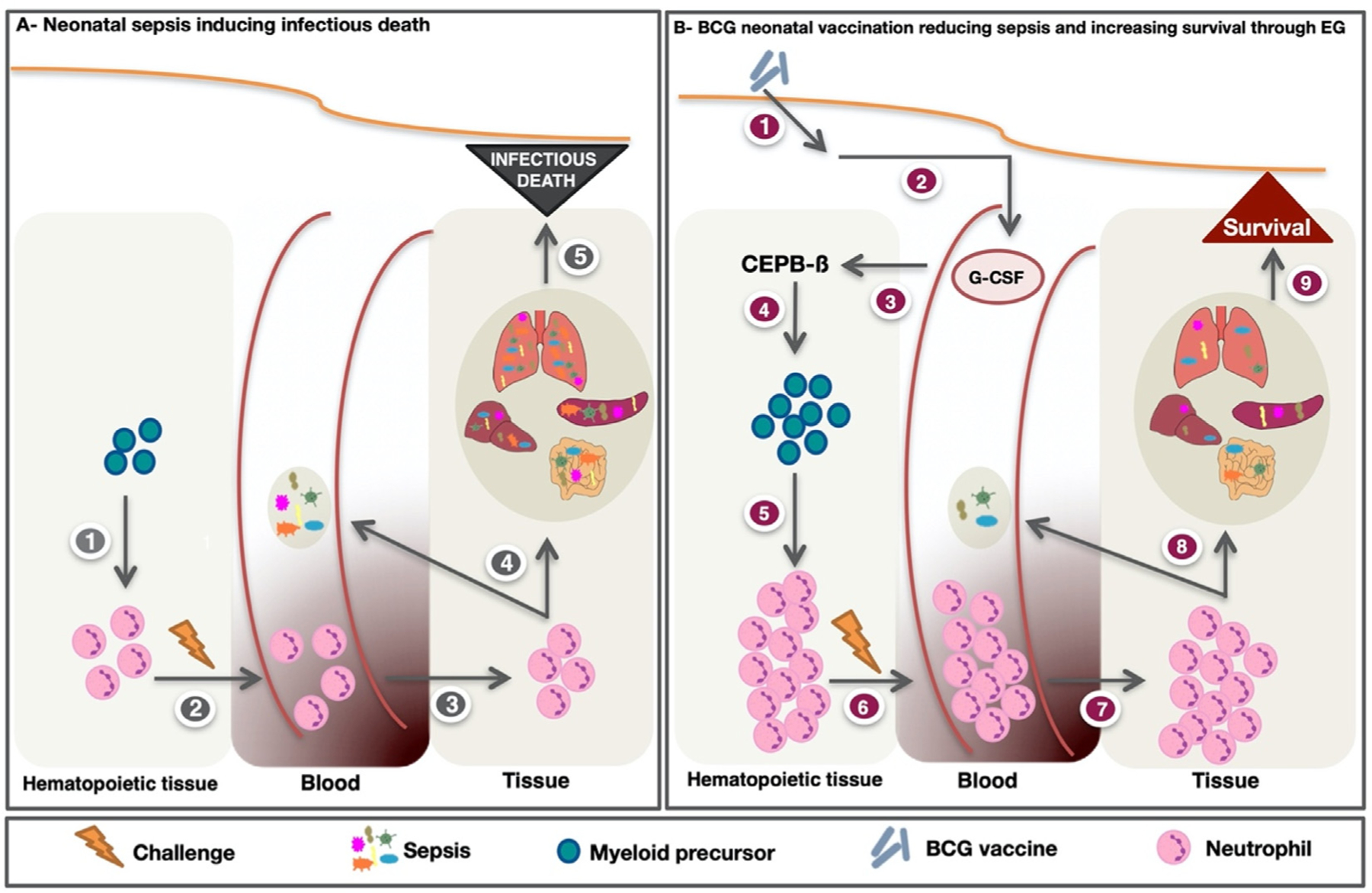
BCG induced emergency granulopoiesis protects newborns from sepsis. (**A**) Control mice are in steady-state granulopoiesis with Granulocyte-Macrophage Progenitors (GMP) differentiating to a resting level of neutrophil production (step 1). Upon challenge (2) the mature neutrophils mobilize to peripheral blood and tissues (3) to combat infection (4). Insufficient neutrophil numbers do not control the infection, making the mice prone to death from polymicrobial sepsis. (**B**) Mice subcutaneously vaccinated with BCG (step 1) produce G-CSF; this G-CSF plasma spike can be measured within hours after vaccination (step 2). This induces emergency granulopoiesis and CEBP-β transcription in the spleen (3), resulting in GMP expansion (4). GMPs multiply and differentiate into an expanded mature neutrophil population (5) that upon challenge (6) mobilize to combat infection (7). The increase in neutrophil numbers enhances the clearance of the systemic bacterial burden (8) improving the chance of survival (9).

BCG at birth protects from all-cause mortality within days of vaccination [6], a period of vulnerability when early-onset neonatal sepsis is a dominant cause of potentially preventable fatal infection [8,9]. Importantly, the randomized clinical trials demonstrating beneficial NSE in the immediate newborn period vaccinated newborns upon discharge from the maternity ward, and excluded newborns unlikely to survive less than 24 h. The mortality rate ratio of BCG vs no BCG was 0.57 (0.35–0.93) for infectious, and 1.20 (0.58–2.49) for non-infectious diseases [6]. With this, it is unlikely that BCG offered protection from non-infectious causes of death like prematurity or birth asphyxia. Neutropenia, defined as <1000 neutrophils/μL blood, is a known risk factor for death from neonatal sepsis [27]. Even in adults, the duration and degree of neutropenia are known to impact the course and outcome of infections [28]. During the phases of acute neutropenia, the reduced number of neutrophils available to combat infection could be further complicated by impairment of neonatal neutrophil function. Compared to adults, neonatal neutrophil function has consistently been described as impaired for chemotaxis, and in release of reactive oxygen species (ROS) or anti-microbial peptides [29]. Other neonatal neutrophil functions, including phagocytosis, and neutrophil extracellular trap extrusion, have been described as impaired, equivalent, and sometimes higher than adult responses, dependent on the stimulus and conditions used [29]. Previous work has demonstrated that enhancing neutrophil function in newborn animals using lipopolysaccharide was associated with enhanced protection from polymicrobial sepsis [30]. We recently demonstrated that newborn BCG vaccination initiates an emergency granulopoiesis response (mediated by transcription factor CCAAT/enhancer-binding protein beta; CEBP-β) which was directly responsible for providing protection against polymicrobial sepsis through a BCG-augmented increase in neutrophil numbers, with no evidence for alterations in the neutrophil function (Fig. 1) [17]. The ability of BCG to amplify neutrophil numbers has previously been observed in adult mice and humans as beneficial [31–34].

### 2.2. Respiratory tract infections

BCG reduces the risk for respiratory tract infections and pneumonia in older adults [35–37]. This supports the hypothesis that BCG may reduce the risk for severe COVID-19 disease that is currently being evaluated in several studies [38–40]. BCG also reduces the risk for lower respiratory tract infection (LRTI) in early infancy [41], and specifically the risk of respiratory syncytial virus (RSV) [11]. The beneficial impact of BCG on respiratory tract infections could also involve neutrophils, since neutrophils play important roles in host defences against both bacterial and viral respiratory tract infections. While the role of neutrophils in bacterial infections has been the best studied, neutrophils are also recognized for their role in viral host defence mechanisms and have been associated with both improved and worse outcomes of viral respiratory disease. By augmenting the activities of T cells, neutrophils promote clearance of viruses [42,43], while also reducing immune damage caused by excessive T cell responses [44]. Conversely, increased disease pathology in severe adult influenza has been associated with neutrophils [45]. Emerging data describing the immune response to COVID-19 also indicates increased neutrophils in the lung early, but diminished levels late in the infectious course [46]. And their role in disease pathology is still being deciphered, but evidence is emerging that heightened neutrophil responsiveness promotes disease severity, and that inhibiting neutrophil effector mechanisms can be beneficial [47–49]. However, inhibiting neutrophil activity has also demonstrated no effect on COVID-19 severity [50]. And preliminary findings from clinical trials have failed to show a beneficial effect of BCG vaccination against COVID-19 [51]. With this, it is still unclear what role neutrophils play in COVID-19 and whether BCG vaccination can impact this. For bacterial respiratory infections, the impact of neutrophils on the host response is in part determined by the content of their granules. For example, neutrophils that produce and release lactoferrin contribute to an anti-inflammatory environment by inhibiting ROS production [52] and sequestering lipopolysaccharide (LPS) [53]. We have observed that BCG can result in greater neutrophil recruitment to the lung, associating with protection, and importantly not causing damage [17]. Evidence from other animal models of mycobacterial infection have also demonstrated that BCG vaccination can also result in increased recruitment of neutrophils to the lung, even months later [54].

Lastly, neutrophils have been shown to be necessary for BCG to induce beneficial NSE against *M. tuberculosis* (*Mtb*) infection in mouse models. Specifically, mice were protected against *Mtb* infection even in the absence of adaptive immunity so long as neutrophils were present at the time of BCG administration. Importantly, this beneficial impact on long term protection was lost when neutrophils were depleted at the time of vaccination, but present at the time of later challenge with *Mtb* [55,56]. This suggests that neutrophils may not only serve as effector cells in mediating NSE but are instrumental in orchestrating an entire downstream network of NSE. The underlying mechanisms must be further studied.

### 2.3. Malaria

Observational studies indicate that BCG decreases the risk of both symptomatic and asymptomatic malaria infection among African children [12] and in Guinea-Bissau, the beneficial effect of receiving BCG-at-birth was strongest in the malaria season [57]. These effects were long-lived, in that children who received BCG in the first month of life still had a reduced risk for malaria infection up to 5 years later. Similarly, infants that had developed a BCG scar were less likely to die of malaria over the following year [10]. A role for neutrophils in this protection could be deduced from animal models [58,59], where neutrophil mobilization during malaria infection can be more readily assessed [60,61]. But while immature neutrophil (band) counts indicative of increased granulopoiesis and neutrophil-to-lymphocyte ratios differed between cases of severe malaria (SM) and uncomplicated malaria (UM) [61], absolute neutrophil counts (ANC) correlated with parasite load only in some studies, not all [62 63]. A direct relationship between peripheral blood neutrophil counts and malaria infection outcome thus has not been established in humans. But differences in neutrophil functions have been found associated with outcomes of human malaria infection. Specifically, improved outcomes from malaria infection have been associated with increased ROS release from human neutrophils [64]; the role of NETosis and phagocytosis are less clear [65].

## 3. Neutrophils in BCG-mediated NSE for non-communicable disease

### 3.1. Bladder cancer

BCG has been used to treat bladder cancer for several decades [66], with solid evidence that neutrophils are central to treatment success [67–69]. Following BCG treatment, neutrophils are recruited to the site of urothelial bladder cancer where neutrophil extracellular trap formation leads to cytotoxicity against cancerous cells inducing apoptosis [67–69]. Their role in the anti-tumour response is unknown.

### 3.2. Autoimmune diseases

BCG vaccination has been associated with reduced risk for multiple autoimmune diseases, including type 1 diabetes (T1D) [70] and multiple sclerosis (MS) [71–73]. While the mechanistic role of NETosis in autoimmune disease pathogenesis is still being investigated, increased capacity for NETosis has been identified as a hallmark of autoimmune disease, but this remains an association. Specifically, elevated levels of NET proteins were found in the serum of MS patients [74], and neutrophils from T1D patients were primed for increased NET production [75,76]. In host defence, neutrophils produce NETs depending on the size of the microorganism, and are known to produce NETs in response to aggregates of microbes such *M. bovis* and *M. tuberculosis* [77,78]. Importantly, NETs were formed in response to bacterial aggregates, but not single cells that could be efficiently phagocytosed [78], and were not always able to kill the mycobacterial cells [77]. Hence, it is possible that BCG vaccination promotes recruitment of neutrophils with priming for NETosis for extended periods, contributing to restored homeostasis in autoimmunity.

## 4. The importance of study design in determining the role of neutrophils in NSE

**Fig. 2.**
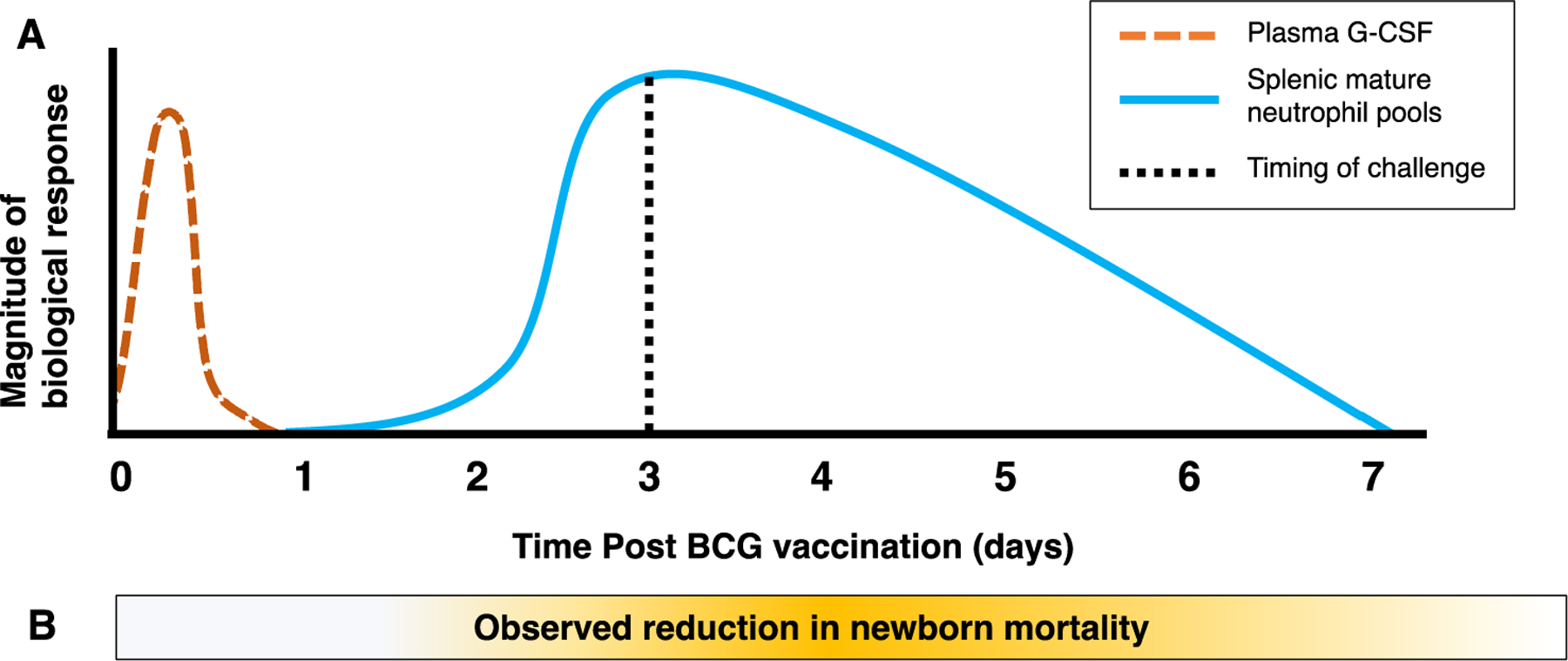
Kinetics of BCG-mediated NSE in the newborn period in animal and human data. **A.** In animal models, BCG vaccination induces rapid production of G-CSF, peaking within 12 h of vaccination (red dotted line), resulting in a peak increase of splenic mature neutrophil pools 3 days following vaccination, and a gradual decrease over the first week of life (solid blue line). Animals are especially well-protected from polymicrobial sepsis when splenic neutrophil pools are at or close to their peak (dotted black line), as these neutrophils rapidly mobilize if mice are challenged. **B.** The beneficial NSE offered by BCG to human newborns were most readily detected between three- and five-days following vaccination (yellow shading).

Identifying responsible biological mechanisms, or establishing causality in place of plausibility, necessitates rational experimental design that is first informed by epidemiological data from humans, probed using animal models or *in vitro* systems, and with the identified mechanisms likely responsible for the effects then subsequently confirmed to be at play in humans. Uncovering mechanisms of BCG-mediated NSE is no exception. Given the dynamic state of immune development in early-life [79,80], coupled with moving targets of infectious exposures [80,81], identifying the *specific* mechanism must be matched to the epidemiological phenomenon in question. Such was the case for identifying the underlying mechanisms of BCG’s protective effects on newborn sepsis. The first randomized trial of BCG for low-birth-weight infants found that protection occurred already within three days of randomization [7] which was deemed as unlikely at the time [82]. A pooled analysis of three randomized trials of BCG given to low-birth-weight infants further supported this initial observation, whereby the reduced risk for mortality occurs in the early neonatal period and within days of BCG receipt [6]. While the primary endpoint of the first two trials was all-cause mortality by one year of age and the first month of life in the third, the mortality rate ratio between BCG-vaccinated and unvaccinated newborns was greatest 3–5 days following randomization, compared to 1–2, or 6–28 days [6,83]. Following these findings, the laboratory investigation, including timing and challenge model, was designed with these trials was designed in mind. The investigation identified emergency granulopoiesis as the likely responsible mechanism, whereby newborn animals are protected from lethal sepsis three days following BCG, but with protection waning within two weeks of vaccination, coinciding with the human data (Fig. 2). Importantly, adult animals were not protected [17]. Hence, experimental design mimicking human observations were crucial in identifying the responsible mechanism for the immediate beneficial effect of BCG on neonatal outcomes. Equally important to emphasize are the limitations of murine models to study complex immunological phenomena that are often confounded by interfering factors in human studies. For example, no sex difference and no effect of maternal priming was identified in the murine granulopoiesis model, while evidence from human trials suggest the influence of both [6,13,84]. Murine models do however provide the opportunity to directly evaluate potential confounders for their interference with BCG-mediated NSE. For example, newborn feeding practice impacts on the risk for death in the first week of life [85]. However, it is unknown how a newborn’s nutritional status impacts on BCG-mediated NSE. Given the known influences of newborn energetic constraints on host defence [86], the impact of newborn nutrition and metabolism on BCG-mediated NSE, including capacity to mount an emergency granulopoiesis response, warrant further investigation. In murine models, feeding practices can be better controlled and aligned, thus reducing the impact of nutritional status on BCG-mediated NSEs.

### 4.1. Limitations of BCG-mediated NSE

While neutrophils can assume diverse roles in the host response to infection aside from their roles in bacterial clearance, enhanced neutrophil function is not expected to offer an advantage in all contexts. And while the long-term effects of BCG on neutrophil phenotype are only emerging, understanding the contexts where BCG does not offer protection will allow prediction of where BCG-mediated NSE acting through augmenting neutrophil function may be beneficial or detrimental. For example, while BCG may offer beneficial NSE against malaria, it was recently shown that a disease tolerance phenotype is beneficial for cerebral malaria specifically [87], meanwhile BCG vaccination was associated with long-lived changes in neutrophil function indicative of enhanced antimicrobial and inflammatory potential [18]. With this, mechanistic studies evaluating BCG-mediated NSE must carefully consider different clinical models. The immediate effects of BCG on emergency granulopoiesis coincided with an increase in both mature and immature neutrophil pools, offering protection from polymicrobial sepsis. Meanwhile, there was no benefit afforded against sterile inflammation induced by a lethal dose of LPS three days following vaccination [17]. While insufficient immune phenotyping was performed to identify these as granulocytic myeloid-derived suppressor cells, this does demonstrate that the presence of these cells had no effect on mitigating systemic inflammation, as has been shown to be a property function of neonatal G-MDSC in the literature [88,89]. Importantly, altering neutrophil function has opposing effects during sterile versus bacterial sepsis, whereby increased neutrophil lifespan is beneficial for bacterial, but detrimental for sterile sepsis [90]. Given the recent finding demonstrating that BCG vaccination results in long-term changes to neutrophil function [18], it is important to evaluate whether this can also increase susceptibility to sterile inflammation weeks following BCG vaccination. Further, while BCG vaccination of human newborns decreased the risk for infectious death in the early newborn period, there was a trend towards increased risk for non-infectious causes of death during that time [6]. While the unknown causes of death were not well characterized, they likely included birth complications, respiratory insufficiency, and prematurity. The roles of BCG on neutrophil function and global NSE should be considered closely in these contexts.

## 5. Outlook

BCG benefits the vaccinee far beyond protection from tuberculosis, challenging the ‘one vaccine for one pathogen’ dogma [91]. Innate immune memory (trained immunity) is one of the mechanisms proposed, challenging the dogma of immune memory as the sole proprietor of adaptive immunity [16]. Rapid induction of emergency granulopoiesis by BCG as central to BCG’s protection from death in newborns with sepsis challenges the dogma of the time a vaccine requires to provide protection [5,17]. Central to all these dogma-challenging revelations now emerges hematopoiesis, and in particular the generation of neutrophils [17,33,34]. Fully embracing these challenges is already inducing a paradigm shift [92] that is poised to bring much needed ‘non-specific’ insight to not just vaccinology and host defense but human health far beyond.

## Abbreviations:

ANC: Absolute neutrophil counts
BCG: Bacillus Calmette-Guerin
CEBP-β: CCAAT/enhancer-binding protein beta
G-CSF: Granulocyte colony stimulating factor
GMP: Granulocyte-Macrophage Progenitor
LPSL: ipopolysaccharide
LRTI: Lower respiratory tract infection
Mtb: Mycobacterium tuberculosis
MS: Multiple sclerosis
NSE: Non-specific effects
ROS: Reactive oxygen species
RSV: Respiratory syncytial virus
SM: Severe malaria
T1D: Type-1 diabetes
UM: Uncomplicated malaria
